# Strengthening Biomarker Research in Canadian Cancer Clinical Trials: A Pathology-Focused White Paper

**DOI:** 10.3390/curroncol33060347

**Published:** 2026-06-09

**Authors:** David F. Schaeffer, Jennifer Chan, Jason Morin, Marie-Christine Guiot, George M. Yousef, Catherine J. Streutker, Harman Sekhon, Madeline Fitzpatrick, Shakeel Virk, Alexander Wyatt, Alan Spatz, Lois Shepherd, Jonathan M. Loree, Mary Kinloch

**Affiliations:** 1 Department of Pathology and Laboratory Medicine, University of British Columbia and Vancouver General Hospital, Vancouver, BC, Canada; 2 Department of Pathology and Laboratory Medicine, University of Calgary and Arnie Charbonneau Cancer Institute, Calgary, AB, Canada; 3 Department of Pathology and Laboratory Medicine, University of Manitoba and Shared Health Manitoba, Winnipeg, MB, Canada; jmorin3@sharedhealthmb.ca; 4 Department of Pathology, Montreal Neurological Institute and McGill University, Montreal, QC, Canada; 5 Department of Laboratory Medicine and Pathobiology, University Health Network and Department of Laboratory Medicine and Pathology, University of Toronto, Toronto, ON, Canada; george.yousef@uhn.ca; 6 Department of Pathology, Unity Health Toronto—St. Michael’s Hospital and Department of Laboratory Medicine and Pathology, University of Toronto, Toronto, ON, Canada; 7 Department of Pathology and Laboratory Medicine, The Ottawa Hospital, University of Ottawa, Ottawa, ON, Canada; 8 Department of Pathology, Eastern Health, Memorial University, St. John’s, NL, Canada; 9 Canadian Cancer Trials Group (CCTG), Queen’s University, Kingston, ON, Canada; 10 BC Cancer and Department of Urologic Sciences, University of British Columbia, Vancouver, BC, Canada; 11 Department of Pathology, McGill University and Department of Pathology, Jewish General Hospital, and Lady Davids Institute for Medical Research, Montreal, QC, Canada; 12 Department of Pathology and Molecular Medicine, Queen’s University, Canadian Cancer Trials Group, Queen’s University, and Kingston Health Sciences Centre, Kingston, ON, Canada; 13 BC Cancer and Department of Medicine, University of British Columbia, Vancouver, BC, Canada; jonathan.loree@bccancer.bc.ca; 14 Department of Pathology and Laboratory Medicine, University of Saskatchewan and Saskatchewan Health Authority, Saskatoon, SK, Canada; mary.kinloch@saskhealthauthority.ca

**Keywords:** pathology, biomarkers, clinical trials, biospecimen attrition, translational oncology, digital pathology, tumor tissue, consent and ethics governance, Canadian Cancer Trials Group, precision oncology

## Abstract

Pathology is foundational to biomarker-driven and translational oncology research, yet systemic barriers limit full pathology engagement in Canadian cancer clinical trials, compromising the tissue-based questions such trials are designed to answer. This commentary and white paper synthesizes the perspectives of a national pathology working group convened at the 2025 Canadian Cancer Trials Group (CCTG) Annual General Meeting with a descriptive internal audit of the CCTG Tumour Tissue Data Repository (TTDR), in which biospecimen attrition was tabulated at the patient level by disease site; no inferential testing was performed. The TTDR data revealed substantial attrition across disease sites, with no tissue submitted for 32–44% of patients and slides submitted in place of formalin-fixed paraffin-embedded blocks for up to 51% of cases, reflecting persistent misalignment between protocol expectations and laboratory capacity. From these observations, five interrelated gaps were identified—in trial design, funding and resourcing, digital pathology infrastructure, academic recognition, and knowledge translation around consent and ethics governance. Five corresponding strategies are proposed to align research demands with pathology capacity, reduce attrition, and strengthen biomarker-driven trials. As a consensus- and experience-driven analysis rather than a systematic review, these recommendations are intended to frame a national conversation and a starting point for prospective evaluation.

## Simple Summary

Pathology is essential to modern cancer clinical trials, especially those that use tumour tissue to decide who can join, what treatment they receive, or whether the treatment is working. However, internal data from the Canadian Cancer Trials Group (CCTG) show that in many trials, tissue samples are either not sent in at all or sent as slides instead of the preferred tissue blocks—sometimes affecting 30–50% of patients depending on the cancer type. This means valuable research questions cannot be fully answered. To investigate the causes and identify solutions, a national working group of Canadian pathologists was convened. They identified five main gaps: pathologists are rarely included when trials are first being designed, current funding does not adequately support the extra work involved, digital pathology infrastructure is limited, pathologists’ contributions to trials are not formally recognized for career advancement, and there is confusion about consent and ethics rules for sharing tissue. The paper proposes five practical strategies to address these gaps, including bringing pathologists into trial design from the start, exploring flexible funding models, building digital pathology infrastructure, creating a way to formally credit pathologists who contribute, and improving education about national consent and ethics policies. The goal is to make sure tissue samples are collected and used effectively, so that Canadian cancer trials can deliver better answers for patients.

## 1. Executive Summary

This paper is a commentary and white paper synthesizing the perspectives of a national pathology working group and an internal descriptive analysis of the Canadian Cancer Trials Group (CCTG) Tumour Tissue Data Repository (TTDR); it is not a formal systematic review or prospective study, and the recommendations presented below should be interpreted in that context.

Pathology is central to the delivery of biomarker-driven and translational research in oncology, yet systemic barriers currently limit full engagement in Canadian cancer clinical trials. Internal analysis of the Canadian Cancer Trials Group (CCTG) Tumour Tissue Data Repository (TTDR) demonstrates substantial biospecimen attrition across multiple disease sites. The TTDR figures cited in this paper are derived from a descriptive internal audit of CCTG-sponsored trials, in which the proportion of enrolled patients with no biospecimen submitted and the proportion submitted as unstained slides in place of formalin-fixed paraffin-embedded (FFPE) blocks were tabulated by disease site at the patient level; no inferential statistical testing was performed, and the figures should be interpreted as a high-level operational summary rather than a hypothesis-driven analysis. For example, 44% of hematologic malignancy cases had no tissue submitted, 51% of genitourinary cases were limited to slide submissions rather than FFPE blocks, and 37% of breast cancer cases lacked specimen submission altogether (full disease-site breakdown provided in Table 1). These findings reveal a persistent misalignment between protocol expectations and the operational capacity of most Canadian pathology laboratories.

From these observations, five key gaps emerge that currently limit pathology engagement in Canadian cancer clinical trials: (1) a **trial-design gap**, where pathology expertise is rarely incorporated during protocol development, resulting in tissue requirements that are operationally unrealistic; (2) a **funding and resource gap**, where current per-case reimbursement does not consistently support the dedicated personnel and infrastructure needed for research-related tissue handling at higher-volume sites; (3) an **infrastructure gap**, with limited national capacity for digital pathology to support remote review and reduce physical block transfer; (4) an **academic recognition gap**, with no consistent mechanism to formally capture and credit local pathologist contributions to trial activities; and (5) a **knowledge-translation gap** regarding consent and ethics governance, where perceived medico-legal risk and incomplete dissemination of nationally harmonized policies discourage tissue release. The five strategies proposed in this paper map directly to these five gaps and are developed in detail in Section 2.1, Section 2.2, Section 2.3, Section 2.4 and Section 2.5.

Pathology departments are predominantly structured to support clinical diagnostic workflows rather than research activities. As a result, participation in research protocols is often constrained by limited personnel, inadequate logistical infrastructure, and absence of standardized academic and financial reimbursement for contributions. Institutional restrictions on the release of FFPE blocks, coupled with perceived medico-legal risks associated with the transfer of diagnostic tissue and linked data, further exacerbate attrition. In certain instances, insufficient diagnostic tissue remains after required clinical testing, creating direct competition between research requests and patient care needs, which take priority.

To address these challenges, CCTG convened a national working group of academic and clinical pathologists during the 2025 CCTG Annual General Meeting to identify challenges and opportunities to strengthen oncology research in Canada. This white paper integrates TTDR data with the working group’s analyses to generate a set of evidence-informed recommendations, to serve as an example of challenges facing oncology research. These barriers exist in both academic and industry-sponsored trials and addressing current gaps will ensure a strong oncology research ecosystem in Canada. Proposed strategies include: (i) systematic integration of pathology expertise during trial design (Section 2.1), (ii) development of sustainable operational funding models (Section 2.2), (iii) implementation of digital pathology infrastructure (Section 2.3), (iv) establishment of transparent frameworks for academic recognition (Section 2.4), and (v) harmonization, standardization, and knowledge translation of consent and ethics procedures (Section 2.5).

Collectively, these interventions are designed to align research demands with pathology capacity, reduce biospecimen attrition, and enhance the reproducibility and impact of biomarker-driven clinical trials in Canada.

## 2. Recommendations

### 2.1. Engagement of Pathologists in Trial Design and Biospecimen Collection

TTDR data underscore the imperative for early integration of pathology expertise in clinical trial design, particularly for studies involving tissue-based endpoints, like pathologic complete response rate [1,2]. To address this need, it is recommended that all such trials formally include a pathologist or pathology-trained investigator as a core member of the trial development team. Although the involvement of a surgical pathologist upfront in the writing of the trial protocol may not be sufficient to address cultural and resource-related barriers, this individual would contribute to the design and authorship of pathology manuals, assess the operational feasibility of biospecimen collection strategies, provide input on biomarker selection and analytical platforms, and help mitigate site-specific barriers to tissue acquisition and submission [3].

One of the most important points of influence for pathology is early in trial design, particularly when the biomarker component is integrated or integral. In these cases, pathology involvement ensures that tissue collection expectations are feasible, clearly justified, and operationally realistic. When a biomarker is integral, participation typically requires providing the tissue to enroll patients—thus, compliance is high. When a biomarker is integrated, and the analysis is part of the trial workflow, sample submission is generally not a barrier.

However, in trials where biospecimen collection is for future, unspecified correlative research, particularly involving archival paraffin tissue, many sites are increasingly reluctant to release material. This reluctance is due in part to uncertainty over how samples will be used, the long delays before analysis (sometimes a decade later), and growing pressure to preserve limited diagnostic material for clinical care. Pathology input at this stage is essential to flag when the ‘asks’ for banking are ambitious or impractical, and to balance scientific goals with operational realities.

A further consideration, even in trials where biospecimen collection is not mandatory for participation, is that protocols frequently request recently acquired (fresh) tissue rather than accepting archival material. For many patients, undergoing an additional biopsy procedure that offers no immediate personal clinical benefit is burdensome, and depending on tumour location and biopsy modality (e.g., image-guided core biopsy of a deep visceral lesion, transbronchial sampling, or repeat surgical biopsy) may be technically difficult, carry non-trivial procedural risk, or simply be impractical. The result is that these “optional” correlative collections often have low real-world uptake, despite high enthusiasm at the protocol-design stage. Early pathology input can help adjudicate when an archival FFPE block would yield equivalent scientific information, when a fresh biopsy is truly justified by the biology being interrogated, and when the patient-level burden of a repeat procedure should be reflected in informed consent and recruitment expectations. Aligning tissue requirements with what is realistically obtainable from the target patient population—rather than what would be scientifically ideal in the abstract—is, in our experience, one of the higher-yield contributions that a pathologist can make during trial development.

Although biobanking strategies were discussed at recent CCTG meetings, including options such as site-retained samples with centralized tracking, these operational models fall outside the scope of this white paper, which focuses on biomarker design strategy rather than biobanking infrastructure. These may be options to consider for academically run trials; however their implementation in industry sponsored trials would be more difficult.

The inclusion of pathology expertise has demonstrated clear value in prior studies—exemplified by the CCTG EN.11 trial, a trial where biomarker classification was required for patient enrollment and where pathologist involvement during trial development directly informed both the pathology manual and eligibility criteria, contributing to improved trial execution [4]. These findings support the routine incorporation of pathology professionals into trial leadership to enhance scientific rigor, logistical alignment, and overall trial success.

**Pros:** Involving pathologists early ensures that tissue collection requirements are realistic and clearly justified. It improves biomarker integration, enhances the scientific quality of trial protocols, and builds trust with pathology departments, which can increase compliance with sample submission.

**Cons:** This approach may lengthen trial development timelines and adds workload for pathologists who are already stretched thin. If not coupled with resources or recognition, the involvement risks being more symbolic than impactful.

### 2.2. Standardized Compensation and Operational Funding with Feedback Metrics

Preferred funding models vary across pathology departments, and there is no single approach that suits every site. Per-case reimbursement, which currently predominates in trial budgets held by Clinical Research Organizations (CROs) and pharmaceutical sponsors, is administratively straightforward, scales transparently with volume, and is favoured by many department chairs precisely because it is easy to track and reconcile. At the same time, working group discussions identified that for higher-volume academic sites supporting numerous concurrent trials, per-case reimbursement may not adequately cover the fixed costs of dedicated research personnel, sample handling infrastructure, and quality oversight; in these settings, some departments have expressed interest in annualized or global operational funding to support stable staffing for research-related tissue handling. A hybrid approach—maintaining per-case reimbursement as the default while making annualized operational support available to sites that exceed a defined volume threshold and meet agreed deliverables—may best accommodate this heterogeneity. We acknowledge that any shift away from per-case funding represents a significant change to current practice and would require careful consideration, including the recognition that funding all sites equally is unlikely to be equitable when case contributions differ by orders of magnitude (e.g., one site contributing over 100 cases per year versus another contributing only one).

Furthermore, most clinical trial funding is built around per-case reimbursement through specific budgets held by Clinical Research Organizations (CROs) or pharmaceutical companies. Transitioning to a centralized or pooled funding model would require structural changes. If funding were to come from a broader source—such as a provincial cancer agency (such as BC Cancer or Ontario Health-Cancer Care Ontario, which administer cancer services at the provincial level in Canada) or an overarching national pathology research operational budget—it would be critical to define deliverables and performance metrics, including annual case volume, turnaround time for tissue requests, and engagement in trial start-up activities (e.g., pathology review of protocols).

Trial participation should continue to include feedback on diagnostic concordance and quality metrics, and any future funding model should reflect both volume and performance. This would ensure accountability and encourage active engagement from pathology departments in trial processes.

**Pros:** Moving from per-patient reimbursement to sustainable operational funding provides dedicated support for research-related tissue handling. This model encourages consistent engagement across sites, could tie funding to measurable outcomes such as turnaround times and annual trial case volumes (i.e., the number of trial-enrolled cases for which biospecimens are processed and submitted per site, per year), and can more accurately reflect the actual costs borne by pathology departments.

**Cons:** This would require significant structural funding changes, which may be difficult to implement. The approach could create inequities between high-volume and low-volume sites, adds administrative complexity in tracking deliverables, and depends heavily on long-term financial commitment from provincial or national agencies.

### 2.3. Digital Pathology as a Strategic Enabler

Digital pathology (also referred to in the literature as virtual microscopy, whole-slide imaging, telepathology, or, in some Francophone settings, “pathologie numérique”/numerical pathology) offers significant opportunities to streamline trial participation by enabling remote review of histologic material and reducing reliance on physical block transfers for central pathology review [5,6]. While digital slides cannot replace tissue for molecular testing, they are invaluable for diagnostic interpretation, case triage, and eligibility confirmation in trials requiring pathology input. Moreover, recent data have demonstrated the power of AI algorithms based on H&E slides to predict molecular alterations and generate prognostic and predictive classifiers [7,8] making invaluable the utility of whole slide image repositories of trial-related H&E slides.

Although a portion of submitted material consists of slides rather than blocks—sometimes due to institutional policies restricting block release —this reinforces the need for alternative review strategies. Digital pathology can support this need by allowing central reviewers and trial pathologists to access high-quality images remotely without compromising patient sample integrity.

Beyond the established role of digital review in rare-disease cases that require centralized confirmation, several common, day-to-day trial scenarios stand to benefit from digital review of pre-prepared slides. Examples include: (i) **eligibility confirmation** for histology-defined enrollment criteria (e.g., confirming a specific subtype such as triple-negative breast cancer, MSI-high colorectal cancer, or small-cell vs. non-small-cell lung cancer) before randomization, which is currently a frequent cause of screen-failure delay; (ii) **central confirmation of biomarker-based stratification**, such as adjudication of HER2 immunohistochemistry equivocal cases, mismatch repair protein loss, or PD-L1 scoring, where inter-observer variability can affect arm assignment; (iii) **pre-screening of tumour content and adequacy** prior to molecular testing, to flag insufficient tumour cellularity or excessive necrosis before scarce tissue is committed to downstream assays; (iv) **response and toxicity adjudication** on post-treatment specimens, such as central scoring of pathologic complete response after neoadjuvant therapy or grading of immune-related adverse-event biopsies; (v) **multidisciplinary case discussion** at trial team meetings, where remote, simultaneous viewing of slides across sites avoids the delays and risks associated with physical slide circulation; and (vi) **retrospective central review and quality assurance** for ongoing or completed trials, including the construction of curated whole-slide-image cohorts for downstream AI model development. In each of these scenarios, digital review of pre-prepared slides offers a faster, lower-risk, and lower-cost alternative to physical slide or block transfer, while preserving the original diagnostic material at the originating institution.

We recommend that future trials pilot a centralized digital slide repository with standardized scanning protocols, including defined magnification and resolution parameters, in close collaboration with the Canadian Association of Pathologists (CAP-ACP). Development of a digital image upload portal could further facilitate national collaboration and trial efficiency.

**Pros:** Digital pathology reduces redundant staining of subsequent H&Es. The technology supports faster eligibility decisions, enables national collaboration, creates opportunities for AI-based research, and lays the foundation for a scalable infrastructure for future clinical trials.

**Cons:** Implementing digital pathology requires significant upfront investment in whole-slide scanners (both histology/tissue-slide and cytology-slide scanners), IT systems, and secure storage [6]. Standardization across institutions can be difficult, and digital slides cannot replace physical tissue for molecular testing. There is also the risk of widening the gap between better-resourced and under-resourced institutions.

### 2.4. Recognizing Academic Contribution for Local Pathologists

Clinical trial organizations and industry sponsors should support academic recognition for local pathologists who contribute to clinical trials, particularly in trials with biomarker components or extensive correlative studies. One approach is to provide a formal certificate of participation for use in university promotion packages. In addition, while many trials have transparent authorship policies that promote inclusion of local contributors through group authorship models or named acknowledgements, particularly when pathology data is central to the study, the challenge is tracking which pathologists have contributed to trial activities. Currently, there is no formal mechanism for capturing this information.

We recommend that contributing pathologists, after undergoing basic Good Clinical Practice (GCP) training, be added to site delegation logs, mirroring the structure used for clinical investigators. This would create a verifiable record of their involvement and support inclusion in study deliverables. Alternatively, pathology departments could maintain internal trial logs listing the pathologists who reviewed material, selected tissue, or participated in feasibility reviews. These records could then be submitted to the central trial team at study completion for authorship consideration. Standardizing this process across institutions would enhance accountability, ensure academic credit, and foster long-term engagement from pathology teams. However, we recognize that this might create an additional burden on already overworked clinical pathology departments.

Several caveats accompany these recommendations. Reliable tracking systems are required to fairly capture contributions, which adds administrative burden. There is also the risk of “authorship inflation” if inclusion is not managed carefully. Finally, recognition alone may not adequately compensate for the time and effort required, nor address underlying resourcing shortages.

**Pros:** Academic recognition incentivizes engagement, supports career advancement, and fosters stronger collaboration with trial leadership while helping sustain long-term participation.

**Cons:** It adds administrative burden, risks diluting the value of authorship if not carefully managed, and may not fully address resource shortages or uneven participation across sites.

### 2.5. Knowledge Translation of Consent and Ethics Governance

National harmonized comprehensive consent and ethics governance documents are used for clinical trials but are not understood by many laboratories. Consequently, laboratories have concerns regarding liability and ethical implications of releasing specimen tissue. Through discussion at our pathology workshop, the lab concerns were more perceived than actual. Knowledge translation efforts to raise awareness around national updates to tissue governance (here defined as the policies, procedures, and institutional accountability frameworks that govern the collection, storage, release, secondary use, and cross-border transfer of patient tissue and its derived data for research, encompassing consent requirements, ethics board oversight, data-sharing agreements, and chain-of-custody documentation) and patient preferences is needed.

To address concerns from laboratories regarding liability and ethical implications of releasing specimen tissue, generating Canadian-specific education and documentation on current policies and widely used standardized consents and policies would improve acceptability. These templates could include standardized language addressing tissue reuse, molecular analysis, and data de-identification, all designed to protect patient confidentiality and support institutional compliance.

To improve awareness and understanding, a concise resource guide or FAQ outlining the ethical framework and consent process used in its trials, with sample documents available upon request, would be beneficial. Additionally, investigators should consider proactive outreach strategies, such as inviting pathologists to participate in protocol development and ethics planning meetings and encouraging attendance at investigator meetings where tissue handling and consent are discussed.

Engaging directly with the pathology community through presentations at national pathology conferences (e.g., the Canadian Association of Pathologists Annual Meeting) or hosting dedicated oncology trial-related pathology workshops would further promote understanding of trial expectations and strengthen partnerships. Regular communication (with opt in/out option), such as quarterly updates or newsletters to laboratory leadership, could also help bridge this gap.

**Pros:** Standardized consent templates and clear guidance on tissue already in use by co-operative groups helps reduce institutional hesitancy and liability concerns. Knowledge translation is needed to build trust with both patients and institutions, and strengthens partnerships through clearer communication and outreach.

**Cons:** Resistance from local Research Ethics Boards (REBs) or local administrative processes at individual sites could hinder harmonization efforts. Ongoing training and updates will be needed to maintain compliance. Concerns over limited tissue availability may remain unresolved, and the process adds another layer of communication for pathologists already under pressure.

## 3. Limitations

Several limitations of this white paper should be acknowledged. First, this commentary is consensus- and experience-driven rather than the product of a systematic review or prospective study. The recommendations reflect the perspectives of a national working group of academic and clinical pathologists convened at the 2025 CCTG Annual General Meeting, and as such are inherently subjective and may not capture the full range of viewpoints across all Canadian institutions, particularly those of community pathology laboratories, industry trial sponsors, and patient partners, whose input was not formally solicited. Second, the supporting TTDR data reflect CCTG-sponsored trial activity and may not be generalizable to industry-sponsored trials, single-institution studies, or non-CCTG cooperative group trials operating under different protocols, governance frameworks, and incentive structures. Third, the TTDR analyses are descriptive: they quantify attrition (no tissue submitted, slides submitted in place of FFPE blocks) but do not directly attribute causation to any single barrier, and individual site-level practices, case mix, and pre-analytic variables that may influence these figures are not adjusted for. Fourth, recommendations such as transitioning to operational funding models, implementing centralized digital pathology infrastructure, and standardizing delegation logs have not been formally piloted or cost-evaluated within the Canadian context; their feasibility, scalability, and unintended consequences (e.g., inequity between high- and low-volume sites) remain to be tested. Finally, the literature base supporting several of the operational claims is limited, and additional empirical work is needed to quantify the benefit of pathologist engagement regarding trial outcomes and biospecimen yield.

## 4. Future Research and Operational Directions

Several lines of further investigation are needed to translate the proposals in this white paper into measurable improvements in Canadian biomarker-driven oncology research. First, prospective data are needed to quantify the impact of early pathologist engagement during protocol development on biospecimen yield, protocol amendments, and time-to-first-patient enrolled; ideally, this would be evaluated through a pre/post comparison within the CCTG portfolio, using TTDR metrics as objective outcomes. Second, alternative funding models—including hybrid per-case plus annualized operational support tied to defined deliverables (e.g., turnaround time, protocol review participation, diagnostic concordance)—should be piloted at a small number of representative sites, with formal cost-effectiveness analysis to inform broader implementation. Third, the establishment and evaluation of a centralized digital pathology repository, developed in collaboration with the Canadian Association of Pathologists (CAP-ACP), should be prioritized; key research questions include the impact on central review turnaround, the validation of AI-based predictive classifiers on whole-slide images derived from Canadian trial cohorts, and the equity implications for under-resourced institutions. Fourth, operational research is needed on the implementation of standardized delegation logs and contribution-tracking tools for pathologists, with attention to administrative burden, uptake across institutions, and the downstream effects on authorship, academic promotion, and recruitment into trial pathology careers. Fifth, qualitative research—including structured surveys of department chairs, trial pathologists, and Research Ethics Boards—would help characterize the gap between perceived and actual medico-legal risk associated with tissue release, and inform the design of targeted knowledge-translation materials. Finally, mechanisms for sustained engagement with patient partners regarding consent, tissue reuse, and data sharing should be developed and evaluated, recognizing that durable progress on biospecimen access ultimately depends on maintaining public trust.

## 5. Conclusions

Taken together, the five strategies developed in Section 2.1, Section 2.2, Section 2.3, Section 2.4 and Section 2.5 form a single integrated framework: early pathology engagement in trial design (Section 2.1) makes downstream tissue collection feasible; flexible funding models (Section 2.2) ensure that the operational work this generates is sustainably resourced; digital pathology infrastructure (Section 2.3) reduces the friction of physical block transfer and central review; transparent academic recognition (Section 2.4) sustains pathologist engagement over time; and harmonized knowledge translation around consent and ethics (Section 2.5) removes the perceived medico-legal barriers that currently constrain tissue release. Each strategy addresses a distinct gap, but their combined effect is the alignment of research demands with pathology capacity. Pathology is a foundational, not ancillary, component of biomarker-driven oncology clinical trials, yet TTDR data show that substantial biospecimen attrition persists across disease sites in Canadian cancer trials. The five gaps identified in this white paper—in trial design, funding, infrastructure, academic recognition, and knowledge translation around consent and ethics—are interrelated, and addressing any one in isolation is unlikely to be sufficient. The strategies proposed in Section 2.1, Section 2.2, Section 2.3, Section 2.4 and Section 2.5 (early pathologist engagement in trial design, flexible funding models that can include hybrid per-case and operational support, scalable digital pathology infrastructure, transparent mechanisms for academic recognition, and harmonized knowledge translation on consent and tissue governance) are intended to be implemented in a coordinated and stepwise fashion, with priorities adapted to local context. None of these recommendations is offered as a definitive prescription: rather, they are intended to frame a national conversation among CCTG, the Canadian Association of Pathologists, provincial cancer agencies, industry sponsors, and patient partners, and to provide a starting point for the prospective evaluations outlined above. Strengthening the role of pathology in Canadian cancer clinical trials will ultimately require sustained collaboration, dedicated resources, and a willingness to test, measure, and refine these proposals over time, with the shared goal of improving the reproducibility, equity, and clinical impact of biomarker-driven oncology research in Canada.

## Figures and Tables

**Table 1:** TTDR (Tumour Tissue Data Repository) Attrition Summary by Disease Site. Sample submission outcomes by disease and trial site. The table summarizes the proportion of patients for whom no tissue samples were received and the proportion of patients for whom slides were received instead of formalin-fixed, paraffin-embedded (FFPE) blocks, stratified by disease site. The number of patients per disease site is indicated in parentheses.

Disease/Trial Site (Number of Patients)	Proportion of Patients with No Samples Received	Proportion of Patients with Slides Received Instead of FFPE Blocks
Brain (*n* = 730)	10%	43%
Breast (*n* = 30,085)	37%	22%
Gastrointestinal (GI) (*n* = 2536)	36%	33%
Genitourinary (GU) (*n* = 2017)	36%	51%
Gynecologic (GYN) (*n* = 1445)	39%	46%
Head & Neck (*n* = 651)	39%	32%
Hematologic (*n* = 1123)	44%	46%
Lung (*n* = 6186)	32%	25%

## Data Availability

No new data were created or analyzed in this study. Data sharing is not applicable to this article.
